# How can we improve the interpretation of systematic reviews?

**DOI:** 10.1186/1741-7015-9-31

**Published:** 2011-03-30

**Authors:** Andrea C Tricco, Sharon E Straus, David Moher

**Affiliations:** 1 Li Ka Shing Knowledge Institute of St Michael's Hospital, Toronto, ON, Canada; 2 Division of Geriatrics, University of Toronto, Toronto, ON, Canada; 3 Ottawa Methods Centre, Clinical Epidemiology Program, Ottawa Hospital Research Institute, Ottawa, ON, Canada; 4 Department of Epidemiology and Community Medicine, Faculty of Medicine, University of Ottawa, Ottawa, ON, Canada

## Abstract

A study conducted by Lai and colleagues, published this week in *BMC Medicine*, suggests that more guidance might be required for interpreting systematic review (SR) results. In the study by Lai and colleagues, positive (or favorable) results were influential in changing participants' prior beliefs about the interventions presented in the systematic review. Other studies have examined the relationship between favorable systematic review results and the publication of systematic reviews. An international registry may decrease the number of unpublished systematic reviews and will hopefully decrease redundancy, increase transparency, and increase collaboration within the SR community. In addition, using guidance from the Preferred Items for Systematic Reviews and Meta-analyses (PRISMA: http://www.prisma-statement.org/) Statement and the Grading of Recommendations Assessment, Development, and Evaluation (GRADE: http://www.gradeworkinggroup.org/) approach can also be used to improve the interpretation of systematic reviews. In this commentary, we highlight important methodological issues related to the conduct and reporting of systematic reviews and also present our own guidance on interpreting systematic reviews.

Please see Research article: http://www.biomedcentral.com/1741-7015/9/30/.

## Introduction

In *BMC Medicine *this week, Lai and colleagues examined the ability of 95 hospital clinicians, allied health professionals, laboratory technicians, and 35 medical students to accurately generate conclusions from four systematic review (SR) abstracts [1]. SRs are syntheses of relevant research consisting of a clearly formulated question and explicit methods to identify, select, critically appraise, extract, and analyze data (The Cochrane Handbook: http://www.cochrane.org/training/cochrane-handbook). A meta-analysis is a statistical technique to quantitatively integrate the results of included studies and is not always conducted in a SR. Lai *et al. *found that although medical students were better able to decipher the correct conclusion compared to hospital staff, only 30.1% of participants correctly identified both the direction of effect and strength of evidence.

A similar study examined the level of agreement between SR results and reviewers' conclusion statements [2]. Two reviewers independently used a categorization guide to classify SR results and conclusions from a sample of 296 SRs indexed in MEDLINE in November 2004. Conflicts were resolved by discussion or the involvement of a third reviewer. Only moderate agreement between SR results and conclusions was observed (kappa = 0.55; 95% confidence interval: 0.47, 0.64). The results of these two studies suggest that more guidance might be required for interpreting SR results. In this commentary, we highlight important methodological issues related to the conduct and reporting of SRs and also present our own guidance on interpreting SRs.

## Discussion

### Methodological issues related to the conduct and reporting of systematic reviews

Publication bias occurs when "investigators, reviewers, and editors submit or accept manuscripts for publication based on the direction or strength of the study findings" [3]. The impact of publication bias has been widely examined for clinical trials [4-8], for which it has been suggested that studies with statistically positive results and large effect sizes can exaggerate a treatment's effectiveness by 20% [9]. These results highlight the importance of including unpublished studies in SRs. However, unpublished studies are often difficult to locate, especially when funded by private industry [10,11]. Clinical trial registries were developed to surmount issues related to publication bias of clinical research, yet challenges to their use persist [12,13].

In the study by Lai and colleagues, positive (or favorable) results were more influential in changing participants' prior beliefs about the interventions presented in the SRs than negative results [1]. Previous studies have examined the relationship between SR results and the publication of SRs. In a cross-sectional study of 296 SRs indexed in MEDLINE, 36.5% of the overall sample had favorable results [2]. This increased to 57.7% for Cochrane and 64.3% for non-Cochrane reviews with a meta-analysis of the primary outcome. In an international survey of 348 SR authors, 1,405 published (median: 2.0, range: 1 to 150) and 199 unpublished (median: 2.0, range: 1 to 33) SRs were reported [14]. Participants reported that 13 out of 19 of the most recent unpublished SRs for which a meta-analysis was conducted had favorable results for their primary outcome. In another study including 93 published Cochrane reviews, the median time to publication was 1.63 years (range 0.15 to 7.31 years); positive and negative results were not associated with the time to publication [15].

The PRISMA Statement calls for an international registry for SR protocols [16], which is currently under development [17]. An international registry may decrease the number of unpublished SRs and will hopefully decrease redundancy, increase transparency, and increase collaboration within the SR community.

### Guidance on interpreting SR results

In the study by Lai and colleagues, the medical students received a structured and clinically integrated evidence-based medicine course, while the hospital practitioners received an introductory course on evidence-based medicine [1]. The medical students were better able to correctly match the SR abstract with the respective conclusion statement, suggesting that different forms of SR training may have a different impact. Their results imply that systematic reviewers and end users of SRs may benefit from education on conducting SRs, including how to interpret SR results. In addition, enhancing the format of SRs to make them user-friendly may improve the interpretation of SR results [18-20]. Examples of such initiatives include Clinical Evidence (http://clinicalevidence.bmj.com) and the Program in Policy Decision-Making (http://www.researchtopolicy.ca/).

Some systematic reviewers include end users of the review (for example, patients, policy makers, health care professionals) in the SR process [21] or circulate a draft of their discussion to their target audience. These efforts increase the applicability and relevance of SR results and promote adequate interpretation of the results from the different stakeholders' perspectives. Peer review feedback on the interpretation of SR results can also be sought by presenting the SR at a conference. Other approaches include using guidance from the Preferred Items for Systematic Reviews and Meta-analyses (PRISMA) Statement, Grading of Recommendations Assessment, Development, and Evaluation (GRADE), and using a categorization guide to interpret meta-analyses.

The PRISMA Statement provides reporting guidance for SR authors [16] and suggests that systematic reviewers should summarize their main SR findings in a balanced manner, including the strength of evidence for each of the main outcomes. The results should be put into context by considering not only their statistical significance, but also the clinical, political, and resource implications of relevance to patients, healthcare providers, and policy-makers. Limitations of the included studies should be discussed by focusing on the risk of bias (or methodological quality) results. Limitations in the SR process itself should be noted, which can be assessed using tools for appraising SR quality (for example, Assessment of Multiple Systematic Reviews; AMSTAR) [22-24].

GRADE considers four factors in grading the strength of recommendations; quality, benefit versus harm, values and preferences, and resources [25,26]. The quality of evidence is based on study limitations, inconsistency of results, imprecision, reporting bias, and indirectness of evidence. GRADE was originally designed for assessing clinical practice guidelines, yet has gained popularity within the SR community and is endorsed by The Cochrane Collaboration (The Cochrane Handbook). Limitations of GRADE include that it requires training, provides limited guidance for examining non-intervention or non-diagnostic studies, and requires 'scientific value judgments' to be made about a body of evidence, which is often difficult for non-experimental research (for example, observational studies, qualitative studies) [27,28].

If a meta-analysis was conducted, SR authors may also find it useful to use a categorization guide to interpret the results [2,9]. Using the example of a SR examining a particular intervention versus a comparator, favorable results (that is, statistically significant positive effect in favor of the intervention with an associated *P*-value ≤0.05 or a trend towards a positive result that is non-statistically significant, see Text Box) would be classified as a positive finding and the authors would recommend the intervention. If the intervention also had a statistically significant increase in adverse events then the authors may recommend the intervention at the discretion of the patient. The authors may not recommend the intervention if there is a statistically significant increase in serious adverse events. Unfavorable results (that is, statistically significant negative effect in favor of the nonintervention comparator with an associated *P*-value ≤0.05 or a trend towards a negative result that is non-statistically significant) would be classified as a negative finding; hence, the authors would advise against the use of the intervention or not recommend the intervention. A neutral result (that is, effect size between 0.95 and 1.05 and the confidence interval (CI) crosses 1 for dichotomous outcomes or the CI crosses 0 for continuous outcomes) would be classified as a neutral finding and the authors would report no evidence supporting or refuting the intervention's effectiveness. Indeterminate results include whether the SR has more than one primary outcome with different results, the meta-analysis is based on few studies or patients or the SR results are likely affected by bias. In these circumstances, the authors may report insufficient evidence or that more research is required.

## Conclusions

An international registry of systematic review protocols may decrease the number of unpublished SRs and will hopefully decrease redundancy, increase transparency, and increase collaboration within the SR community. The interpretation of SR results may be improved by educating systematic reviewers and end users of SRs, enhancing the format of SRs to make them user-friendly, and including end users in the entire review process. Other approaches include using the PRISMA Statement, GRADE, and a categorization guide for meta-analysis results. Such efforts will increase the applicability and relevance of the SR results and may help to ensure adequate interpretation of the results.

### Text box: Meta-analysis

The outcome used in a meta-analysis is determined by the data obtained from the included studies. Binary (or event) data can be meta-analyzed using odds ratios, relative risk, and the risk difference. Continuous data can be meta-analyzed using the mean difference and standardized mean difference. Other types of outcomes that can be meta-analyzed include hazard ratios (takes time into consideration) and correlation coefficients. Two main types of models used for meta-analysis include the fixed effects model and the random effects model. The fixed effects model does not take between-study variability into account, while the random effects model does [29]. Meta-regression is a statistical tool that can be used to examine how variables of interest are related to the meta-analysis results [30,31]. Bayesian approaches to meta-analyses have also been used [32,33]. Further information on meta-analysis can be found in The Cochrane Handbook.

## Abbreviations

AMSTAR: Assessment of Multiple Systematic Reviews; GRADE: Grading of Recommendations Assessment, Development, and Evaluation; PRISMA: Preferred Items for Systematic Reviews and Meta-analyses; SR: systematic review

## Competing interests

Drs Tricco and Moher published an article on the agreement between systematic review results and conclusion statements. Dr Moher is an author of the Preferred Reporting Items for Systematic Reviews and Meta-analyses. Dr Straus has nothing to declare.

## Pre-publication history

The pre-publication history for this paper can be accessed here:

http://www.biomedcentral.com/1741-7015/9/31/prepub
